# Preface to the Periscope

**Published:** 1825-10-01

**Authors:** 


### Quarterly Periscope

OF

### PRACTICAL MEDICINE;

BEING

### The Spirit of the Medical Journals,

*Foreign and Domestic;*

WITH COMMENTARIES.

### PREFACE.

The Periscopic Department of this Journal has now, we trust, established its claim to the merit of public utility; and we take some credit to ourselves for having been the first, in this Country, to strike out a new path in Periodical Medicine, which, (under various titles) has been since pursued by several cotemporaries on both sides of the Atlantic.* Previously to the establishment of our Periscope, a very large and constantly increasing proportion of medical writings, in the shape of *original communications*, almost entirely escaped the censorship of the press, and were not deemed legitimate subjects for comment or criticism. But such immunity is no longer maintained; and, indeed, we may boldly affirm that this department (of original communications) from its extent and importance, is now of greater interest than the publication of systematic works, or separate treatises. These last are very generally compilations; whereas the detached communications in Medical Journals are more likely to be original facts, observations, or opinions. To bear upon this branch of medical literature, therefore, the Periscope was instituted—with two great objects in view—*first*, to collect the spirit and substance of these widely scattered fragments of science, so as to bring them distinctly under general inspection—and *secondly*, to exercise a mild but independent system of comment, rather than of criticism, with the view of giving currency to their merits, and correction to their errors, without injury or offence to their authors.

If such an object can be effected through the medium of a *Periscope*—and we trust that we have not been hitherto unsuccessful—we think it will be granted, that our labours are of considerable service to the profession at large. To this department of our Journal we cannot conceive a single valid objection. By it we injure no class of the community—neither writers, publishers, nor readers. On the contrary, we benefit them all. The object of a writer must be publicity to his sentiments—and this we give him, in a degree which, from various circumstances, cannot be equalled, or at all events surpassed, through any other channel. This increased publicity stimulates to the communication of original papers in cotemporary Journals—and we have the best means of knowing, that our Periscope every

* The Philadelphia Quarterly Journal has adopted the title, as well as the plan, in its New Series.

day tends to draw forth valuable facts through the aforesaid channels. So far our labours are beneficial, both to the editors and publishers of other periodicals. To the great mass of medical society—the *readers*—we need hardly appeal in favour of our plan. They cannot but see and feel the immense advantages which this new and laborious department has placed within their reach—but they know not the intellectual wear and tear which its construction occasions!

This much we may be allowed to say, that the history of the periodical press in this country—we mean the medical press—presents no epoch when *half* the quantity of practical information was ever conveyed in *double* the space, or for *quadruple* the price of a PERISCOPE. In organizing a department of this kind, therefore, which is now very generally adopted, we think we have "done the state some service"—and we shall not relax our efforts in bringing the said department to its utmost degree of perfection.

To effect this object, we are compelled to reiterate a resolution which has been several times previously announced, but continues still to be imperfectly understood by the public:—we mean the resolution *not to publish original commmunications, except in the Extra-limites, and at the expence of their authors.* Although this determination has been often declared, we daily receive papers which we are obliged to return, and which occasion a loss of time in correspondence that can be but ill spared. We hope that this notice will suffice for the future, and that our practice of reviewing all good papers that appear in other Journals will be a sufficient apology for our declining the insertion of them in any other shape hereafter.

The EXTRA-LIMITES shall always be open to those who deem such a medium desirable; but it must be under the stipulations alluded to above. The quantity of letter-press (beyond that of all other Journals of equal price) which we quarterly devote to our present plan, compels us to rigidly adhere to the resolution now recorded; and we hope that our friends and correspondents will not require further explanation.

